# A Question of Sanity

**Published:** 1886-03

**Authors:** 


### A Question of Sanity.

"No, sir; I haven't seen the will, but I propose to fight it. My uncle was crazier than a loon, and couldn't make a will."

Lawyer Filchem: "But I drew it for him, and know that he bequeaths his entire estate to you."

"Is that so? Then just consider yourself retained to defend the instrument. I propose to protect my dear uncle's memory to the farthest extremity."

